# Inappropriate use of the term “cytotoxicity” in scientific literature

**DOI:** 10.1186/s40199-015-0102-0

**Published:** 2015-02-20

**Authors:** Haji Bahadar, Mohammad Abdollahi

**Affiliations:** Pharmaceutical Sciences Research Center, International Campus, Tehran University of Medical Sciences, Tehran, Iran; Faculty of Pharmacy, Tehran University of Medical Sciences, Tehran, Iran

We would like to draw the attention of the readers to the inappropriate use of term “cytotoxic” as a substitute for “antineoplastic”. This can be noticed in numerous scientific articles, especially those reporting the anticancer properties of certain chemical compounds. For example, there are published studies reporting the anticancer activity of certain drugs/chemicals and the authors have assessed the anticancer activities of some novel drugs against certain target cancers. These investigations have repeatedly involved the term “cytotoxic” instead of “antineoplastic”. For instance, recently a report has been published in DARU by Vosough et al. [1] about the anticancer activities of some compounds. In this study, the derivatives of nonsteroidal aromatase inhibitors (triazole analogues) have been synthesized. Then, the newly synthesized derivatives were assessed for anticancer activity against breast cancer cell lines and the results showed that these newly synthesized compounds possess potent cytotoxic activity. Similarly, there are numerous other research reports published either in DARU or elsewhere. These authors also have reported the anticancer activities of different compounds against various cancer cell lines [2-4]. Nonetheless, these authors have assessed the cytotoxicity as a measure of anti-neoplastic potential of certain drugs, but, reporting the effect as “cytotoxic” instead of either “anticancer” or “antineoplastic” might reduce the interest of readers.

Literally cytotoxic refers to “toxic to living cells” while, antineoplastic means inhibiting or preventing the development and spread of neoplasms. For drugs that control or kill only neoplastic cells, the proper term that could describe this inherent activity of drugs is known as antineoplastic or anticancer activity. Moreover, the physiology of a normal cell is entirely dissimilar to that of cancerous cells or neoplasms, having an excessive quantity of free radicals and subsequent oxidative stress [5,6]. Cytotoxicity is a general term used in toxicological studies describing the effect of particular toxins on cell viability [7]. In that respect, there are many compounds that are cytotoxic but not antineoplastic [8]. About anticancer drugs, data can be gathered using cytotoxicity test methods, but that necessarily be described as the antineoplastic or anticancer activity of that particular drug or agent, rather than stating cytotoxicity of anticancer drugs. Because the term cytotoxicity is an umbrella term involving also other toxins which reduce normal cell viability. In simpler words, we may say that a drug with antineoplastic activity possess cytotoxicity. But any cytotoxic agent does not necessarily possess antineoplastic characteristics.

At the end, we are of the opinion that great care must be exercised while using the term cytotoxicity as an alternative to antineoplastic activity, particularly mentioning the activity of anticancer agents. Also, the National Library of Medicine–Medical Subject Headings (MeSH) http://www.nlm.nih.gov/mesh/MBrowser.html does not suggest the term cytotoxic as a substitute for antineoplastic, but the opposite could easily be seen. Furthermore, MeSH is a very comprehensive controlled scientific terminology helping in proper indexing of the scientific article and books.
